# Supplementary material for: Does educational level predict hearing aid self-efficacy in experienced older adult hearing aid users from Latin America? Validation process of the Spanish version of the MARS-HA questionnaire
Source: PLoS One. 2019 Dec 19;14(12):e0226085. doi: 10.1371/journal.pone.0226085 (PMC6922414; doi:10.1371/journal.pone.0226085)
Supplement: S1 Annex — (DOCX) [file pone.0226085.s001.docx]

**S1. Annex**

**Spanish version of the Measure of Audiologic Rehabilitation Self-Efficacy for Hearing Aids questionnaire (S-MARS-HA)**

**Versión al español del cuestionario de Medición de Autoeficacia de Rehabilitación Audiológica (S-MARS-HA)**

Instrucciones al paciente: Estas preguntas se relacionan con su habilidad de realizar ciertas actividades con audífonos, y además con su habilidad para escuchar en ciertas situaciones. Si usted nunca ha estado en estas situaciones, haga su mejor esfuerzo para suponer que tan bien lo haría. Si usted nunca ha usado audífono responda pensando acerca de su habilidad general para realizar las actividades aquí descritas, como por ejemplo: ¿Qué tan bueno es usted para nombrar la marca y el modelo de los objetos que posee? ó ¿Qué tan bueno es usted en el cambio de baterías o pilas de los objetos que tiene?

Si usted ha visto un audífono, posiblemente pueda o no saber cómo utilizarlo. Quizás ha recibió información acerca de cómo usar el audífono o no. Considerando lo que usted sabe ahora, indique cuanta seguridad tiene para hacer las cosas que aquí se describen. Si cree que no puede hacer la actividad descrita, entonces en la escala de puntuación encierre en un circulo 0% “No puedo hacerlo de ninguna forma”. Si está absolutamente seguro puede hacer la actividad descrita, entonces en la escala de puntuación encierre en un circulo 100% “Seguro que puedo”. Si está un poco inseguro, entonces en la escala de puntuación elija un número entre 0 y 100 que indique que tan confiado se siente de poder realizar la actividad descrita. Los números más altos indican mayor grado de seguridad.

Actividades de práctica: Los siguientes dos ejemplos tienen por objetivo que usted pueda practicar usando la escala de puntuación antes descrita. Por favor, encierre en un círculo el porcentaje que mejor describa su grado de seguridad acerca de cada ejemplo: (1) Puedo levantar un objeto de 4 kilos y medio con facilidad. ¿Con qué seguridad usted puede hacer esto? (encierre el porcentaje en un círculo); (2) Puedo distinguir fácilmente la diferencia entre un objeto de 8 kilos y uno de 9 kilos. ¿Con qué seguridad usted puede hacer esto? (encierre el porcentaje en un círculo).

**Ítems de la versión al español del “Cuestionario de Medición de Auto-eficacia de Rehabilitación Audiológica” (S-MARS-HA):**

1. Puedo colocar la pila en el audífono con facilidad.

2. Puedo sacar la pila del audífono con facilidad.

3. Puedo distinguir entre un audífono para el oído derecho y uno para el oído izquierdo.

4. Puedo colocar de manera correcta un audífono en mis oídos.

5. Puedo sacar un audífono de mis oídos con facilidad.

6. Puedo identificar las diferentes partes de un audífono (micrófono, portapilas, ventilación, parlante, etc.)

7. Puedo manipular de manera correcta todos los controles de un audífono (botones, interruptores y/o control remoto).

8. Puedo hacer que un audífono deje de chirriar.

9. Puedo resolver el problema de un audífono cuando deja de funcionar.

10. Puedo limpiar y cuidar un audífono de manera frecuente.

11. Puedo nombrar la marca y el modelo de un determinado audífono.

12. Puedo nombrar el tamaño de una pila que usa un determinado un audífono.

13. Podría acostumbrarme a la calidad del sonido que entrega un audífono.

14. Podría acostumbrarme a la sensación de tener un audífono en mi oído.

15. Si usara audífonos, podría acostumbrarme al sonido de mi voz.

16. Si usara audífonos, podría entender una conversación con una persona en un lugar silencioso.

17. Si usara audífonos, podría entender una conversación con un grupo de persona en un lugar silencioso.

18. Si usara audífonos podría entender una conversación por teléfono.

19. Si usara audífonos podría entender la televisión.

20. Si usara audífonos podría entender a un expositor en una reunión o presentación.

21. Si usara audífonos, podría entender una conversación con una persona en un lugar ruidoso.

22. Si usara audífonos, podría entender una conversación con un grupo de personas en un lugar ruidoso.

23. Si usara audífono podría entender los avisos entregados a través de parlantes en lugares públicos.

24. Si usara audífono, podría entender una conversación en un auto.
