# Supplementary material for: Does educational level predict hearing aid self-efficacy in experienced older adult hearing aid users from Latin America? Validation process of the Spanish version of the MARS-HA questionnaire
Source: PLoS One. 2019 Dec 19;14(12):e0226085. doi: 10.1371/journal.pone.0226085 (PMC6922414; doi:10.1371/journal.pone.0226085)
Supplement: Attachment — Submitted filename: Plos One S-MARS-HA.docx [file pone.0226085.s003.docx]

Overall, the aim of this paper was to investigate whether hearing aid self-efficacy is related to educational level, selecting a population from a Latin American country on the argument that this population may vary in educational level (which turned out to be true in this sample). The self-efficacy measure is published, well-constructed, and has been applied successfully in several past hearing aid studies on a range of topics. The authors applied a rigorous, well-described translation process to this questionnaire in order to develop a Spanish-language version aiming to maintain the questionnaire’s items and scoring methods. The authors also piloted this translation in a sample of 48 participants to ensure that it was feasible for use, prior to applying it in the current study in a well-justified larger sample. In this sample, the authors performed data collection and analysis to answer the primary aim of the study, relating hearing aid self-efficacy to educational level, among several other factors. Overall, this method is reasonable and the question is of interest in our understanding of factors that relate to hearing aid self-efficacy, which on its own has been shown to impact rehabilitation outcomes. It is not surprising that the authors found a relationship between hearing aid self-efficacy and educational level.

This is a well-conducted study that makes a good contribution to the literature, and may provide guidance to those who provide hearing rehabilitation services or those who develop hearing aid user’s guides for a wide range of populations.

My only concern with this paper is its actual breadth versus its title. With a full translation, feasibility evaluation, and confirmatory factor analysis to not only develop but also to validate the Spanish version of the MARS-HA, this paper has not one but two primary aims. Only the latter is identified in the title.

Minor comments:

Page 6, 2^nd^ paragraph:

I don’t think the translated version can be considered “preliminary” and “final” at the same time.

“Equivalencies” or “equivalence” perhaps rather than “equivalences”

Page 9, bottom:

“hours” rather than “hour”

Page 11, adherence to hearing aids

You have chosen to use a self-report measure of adherence rather than the data log of hearing aid use provided by the hearing aid. Please justify this choice (perhaps reading data logs was not feasible in the context of home visits) and include literature (I know of one 2014 study by Laplante-Leveque et al) on the validity of self-report. Given the large reporting ranges used on the IOI, minor differences between data logs and self report may not have impacted your analyses, but it would be helpful to the reader to acknowledge any limitations of self-report use metrics, either here or in the Discussion.

Page 27

The sentence starting with “The fact that these pairs of items” needs some editing. I think “other than the factor with which they loaded” might be what you meant.

Participants were fitted with hearing aids that did not offer noise management. Why was this unusual fitting profile selected? Is this due to the aids that were available (unlikely) or was it a decision to not provide this? Do you think this explains your low scores on aided listening?

Page 28

The sentence ending with “a better hearing aid self-efficacy” needs some editing. Perhaps “… older adults with higher educational levels exhibited higher hearing aid self-efficacy”.

Page 28, last paragraph

When I first read “readability of the written materials” I initially thought you were referring to the S-MARS-HA but as I read on, I realized you mean the hearing aid instruction manuals. Please clarify, perhaps “readability of the written materials (e.g., hearing aid user’s guide)”.
